# 864. Adolescent Primary Care Provider Knowledge of HIV Pre-Exposure Prophylaxis (PrEP) and Universal Screening at a Midwest Academic Medical Center

**DOI:** 10.1093/ofid/ofab466.1059

**Published:** 2021-12-04

**Authors:** Genevieve Allen, Jamie Riddell

**Affiliations:** 1 University of Michigan Health, Ann Arbor, MI; 2 University of Michigan, Ann Arbor, MI

## Abstract

**Background:**

HIV remains a problem for adolescents with 21% of new infections in the United States in 2018 occurring in youth. In this study we attempted to assess the knowledge of and comfort with pre-exposure prophylaxis and universal HIV testing among adolescent primary care providers affiliated with one academic medical center.

**Methods:**

We conducted a survey of internal medicine/pediatrics, pediatrics, and family medicine residents and attending physicians affiliated with an academic medical center. Data collected included provider prescribing and referring habits for PrEP and information on their universal HIV testing habits. A “test your knowledge” section followed the survey which asked participants to name PrEP medications and to correctly select laboratory monitoring required for PrEP. Correct answers and prescribing resources were provided on completion of the survey.

**Results:**

138 (76%) respondents were aware that PrEP is approved for adolescents. There was no significant difference across specialties or between residents and attendings. 44.8% of respondents felt uncomfortable prescribing PrEP and two thirds had never prescribed PrEP. Reasons for not prescribing PrEP included: not seeing adolescents who qualify (n=80), not having enough training (66), confidentiality concerns (22), forgetting to address PrEP (19), and concern incidence of HIV is too low to recommend PrEP (15). Pediatricians were the least likely to test for HIV with 11% of pediatrician, 32% of internal medicine/pediatric, and 38% of family medicine respondents reported universal HIV testing for patients 15 years and older (p < 0.05). Residents were more likely to test for HIV than attendings (33.3% versus 16%, p < 0.05). 111 participants completed the “test your knowledge” section. 31.5% correctly named two approved PrEP medications. There were 183 responses to the survey (49% response rate).

**Conclusion:**

Adolescent primary care providers are aware that PrEP is FDA approved for adolescents but a gap in PrEP prescribing and HIV testing persists. There remain perceptions that HIV incidence is too low to discuss PrEP and that providers are not seeing patients who qualify. Next steps include developing an institutional PrEP guideline and creating an electronic medical record order set to facilitate PrEP prescribing.

**Disclosures:**

**All Authors**: No reported disclosures

